# Quality and safety actions in primary care practices in COVID-19 pandemic: the PRICOV-19 study in Spain

**DOI:** 10.1186/s12875-024-02391-8

**Published:** 2024-05-13

**Authors:** Alba Gallego-Royo, Inés Sebastián Sánchez, Leticia-Ainhoa Sanz-Astier, Antoni Peris-Grao, Nuria Freixenet-Guitart, Jose Angel Maderuelo-Fernández, Rosa Magallón-Botaya, Bárbara Oliván-Blázquez, Esther Van Poel, Sara Willems, Sara Ares-Blanco, María Pilar Astier-Peña

**Affiliations:** 1 Preventive Medicine, Miguel Servet University Hospital, Zaragoza, Spain; 2 Aragonese Health Service, Aragón, Spain; 3 University of Zaragoza, Zaragoza, Spain; 4 GIBA, Aragon Bioethics Research Group. IIS Aragón, Zaragoza, Spain; 5 RICAPPS. Research Network on Chronicity, Primary Care and Health Promotion, Tenerife, Spain; 6 Aragonese Health Service, Universitas Health Centre, Zaragoza, Spain; 7 Internal Medicine Service, Bellvitge University Hospital, Barcelona, Spain; 8 Catalan Institute of Health, Catalonia, Spain; 9 Castelldefels Health Agents (CASAP). Castelldefels, Catalonia, Spain; 10 Salamanca Primary Care Research Unit (APISAL), Institute of Biomedical Research of Salamanca (IBSAL), Gerencia de Atención Primaria de Salamanca, Gerencia Regional de salud de Castilla y León (SACyL), Salamanca, Spain; 11 Network for Research on Chronicity, Primary Care, and Health Promotion (RICAPPS), Mallorca, Spain; 12 Faculty of Medicine and Health Sciences, Department of Public Health and Primary Care, Ghent University, Ghent, Belgium; 13 Federica Montseny Health Centre, Gerencia Asistencial de Atención Primaria, Servicio Madrileño de Salud, Madrid, Spain; 14 Gregorio Marañón Institute of Biomedical Research, Madrid, Spain; 15 EGPRN, European General Practitioners Network, Maastricht, The Netherlands; 16 Primary Health Care Research Group of Aragon (GAIAP), B21-20R. IIS-Aragón, Zaragoza, Spain; 17 QiT research group., Idiap Jordi Gol i Gudina, Tarragona, Spain

**Keywords:** COVID-19, Primary health care, Healthcare quality, Patient safety, Spain, Europe, PRICOV-19, General practice, Family medicine

## Abstract

**Background:**

Primary Health Care (PHC) has been key element in detection, monitoring and treatment of COVID-19 cases in Spain. We describe how PHC practices (PCPs) organized healthcare to guarantee quality and safety and, if there were differences among the 17 Spanish regions according to the COVID-19 prevalence.

**Methods:**

Cross-sectional study through the PRICOV-19 European Online Survey in PCPs in Spain. The questionnaire included structure and process items per PCP. Data collection was due from January to May 2021. A descriptive and comparative analysis and a logistic regression model were performed to identify differences among regions by COVID-19 prevalence (low < 5% or high ≥5%).

**Results:**

Two hundred sixty-six PCPs answered. 83.8% of PCPs were in high prevalence regions. Over 70% PCPs were multi-professional teams. PCPs attended mainly elderly (60.9%) and chronic patients (53.0%). Regarding structure indicators, no differences by prevalence detected. In 77.1% of PCPs administrative staff were more involved in providing recommendations. Only 53% of PCPs had a phone protocol although 73% of administrative staff participated in phone triage. High prevalence regions offered remote assessment (20.4% vs 2.3%, p 0.004) and online platforms to download administrative documents more frequently than low prevalence (30% vs 4.7%, *p* < 0.001).

More backup staff members were hired by health authorities in high prevalence regions, especially nurses (63.9% vs 37.8%, *p* < 0.001. OR:4.20 (1.01-8.71)). 63.5% of PCPs provided proactive care for chronic patients. 41.0% of PCPs recognized that patients with serious conditions did not know to get an appointment.

Urgent conditions suffered delayed care in 79.1% of PCPs in low prevalence compared to 65.9% in high prevalence regions (p 0.240). A 68% of PCPs agreed on having inadequate support from the government to provide proper functioning. 61% of high prevalence PCPs and 69.5% of low ones (p: 0.036) perceived as positive the role of governmental guidelines for management of COVID-19.

**Conclusions:**

Spanish PCPs shared a basic standardized PCPs’ structure and common clinical procedures due to the centralization of public health authority in the pandemic. Therefore, no relevant differences in safety and quality of care between regions with high and low prevalence were detected. Nurses and administrative staff were hired efficiently in response to the pandemic. Delay in care happened in patients with serious conditions and little follow-up for mental health and intimate partner violence affected patients was identified. Nevertheless, proactive care was offered for chronic patients in most of the PCPs.

**Supplementary Information:**

The online version contains supplementary material available at 10.1186/s12875-024-02391-8.

## Background

**Fig. 1:**
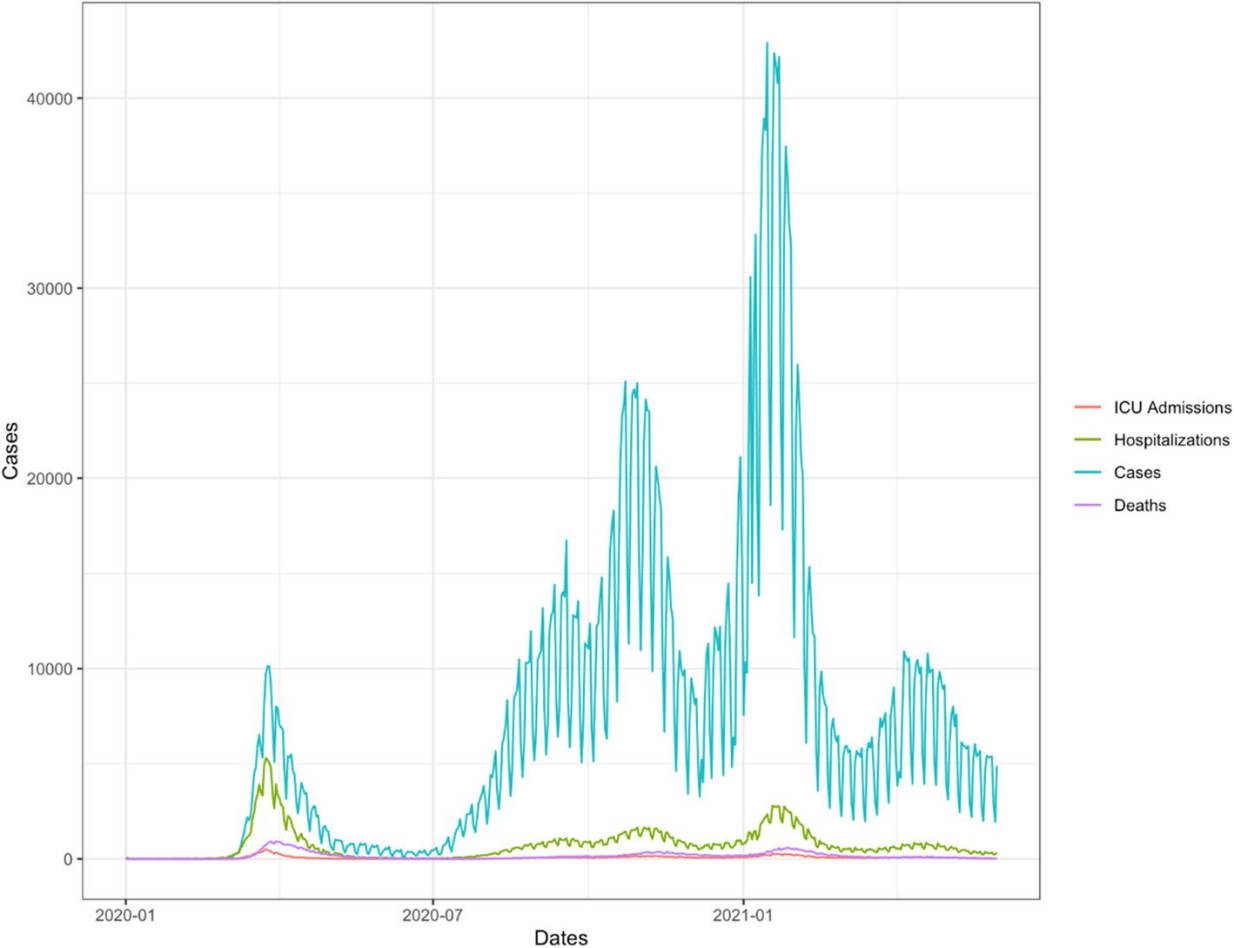
Evolution of cases, hospitalizations, ICU admissions and deaths from COVID-19 from January 2020 till May 2021

Spain is in the top four countries with the highest number of COVID-19 cases in Europe [1]. The country suffered from five pandemic waves by September 2021 (Fig. 1A). Primary health care (PHC) has been a key element in the detection, monitoring and treatment of both patients and their contacts, offering patient-centred care [2, 3]. The exponential increase in healthcare activity during epidemic waves has required a reorganisation and adaptation of PHC [4]. The World Health Organization (WHO) established initial strategies based on a public health and hospital approach, with recommendations that were poorly defined for PHC teams, and subject to the decision of each individual state [5]. The role of PHC was not the same in all countries, with great variability, based on the health system model. Preventive measures and diagnostic testing strategies were established at central level in most of the countries [6].

Several organisations were already warning of the need to strengthen the health system and especially PHC to be able to face future health challenges [7, 8]. During the COVID-19 health crisis, European countries, whose PHC was mainly public, established Primary Care Practices (PCPs) as the key points of health care for COVID-19 cases. However, governments did not increase significantly PHC resources despite of PCPs already had a high workload before pandemic. At the first stage of COVID-19 pandemic, in public provision healthcare services, PCPs had to implement and manage structural changes, and staff and equipment reorganization, without increasing the structural endowment or remuneration improvements. Nevertheless, improvements were easily introduced and funded in those healthcare systems with private provision [6].

Spanish National Health System (SNHS) has almost universal healthcare coverage, mainly funded from taxes and with mainly public provision for PHC. PCPs are the first contact for citizens with the SNHS [9]. The country is organize in 17 Autonomous communities or regions, and there are 17 regional healthcare services. Healthcare professionals are salaried by the public health authority. There is a PCP with a population up to 25,000 inhabitants. PCPs are organized with one family doctor (FD) and a nurse per 1500 to 2000 registered patients. In general, there are a midwife, a social worker, a physiotherapist and sometimes, a mental health professional per PCP.

At national level, Interterritorial Committee for the Spanish National Health System (ICSNHS) coordinates healthcare provision [10] and public health for the 17 regional healthcare services.

PHC provision is free of charge at the point of delivery, except for outpatient pharmaceutical prescriptions. A PCP has a Primary Care Team (PCT). All citizens can get an appointment in the PCP with a family doctor, a nurse, a midwife, a mental health professional or a physiotherapist.

The alarm state was declared in Spain March 15th and lasted until June 21st 2020 [11]. During this period, the Ministry of Health was in charge of protocols and health care organization of the COVID-19 pandemic nationwide. All the regional healthcare services and public health coordination through the sanitary alerts and emergencies coordination centre (CCAES) [12]. ICSNHS met weekly and launched a report to coordinate healthcare along the regions.

During COVID-19 Pandemic, PCP in each region has been providing contact tracing, testing, and treating mild to moderate COVID-19 cases as well as managing COVID-19 sick leave [6, 13, 14]. At the same time, they provided usual care to the registered population too. Most of the regions increased the number of primary care consultations in 2020 compared to previous years, without increasing the number of medical professionals in PCPs [15] (Supplement 1).

Few studies showed the role of primary care during the health crisis, and those studies described that most of patients affected by SARS-CoV-2 were treated in PHC, and only half of the patients were treated in emergency departments required hospital admission [4] In turn, there is wide variability in the performance of complementary tests and referral criteria from the different PCPs, which may be related to the burden of care assumed by PHC teams since the beginning of the pandemic [4].

Therefore, how Spanish public PCPs guaranteed healthcare quality and safety during the first year of COVID-19 pandemic is not very well known. This study [16] aims to determine which PCPs’ characteristics were associated with safe, effective, patient-centred and equitable healthcare, considering the different prevalence of COVD19 pandemic among regions in Spain.

## Methods

### The PRICOV-19 study

The PRICOV-19 study aimed to describe how primary care practices) were organized during the COVID-19 pandemic to guarantee high-quality care; how tasks and roles changed, and the impact of the pandemic on the wellbeing of care providers. Europe PRICOV-19 study aimed to sample between 80 to 200 PCPs per country. One questionnaire should be completed by each PCP, preferably by a FD or by a staff member familiar with the practice organization [10].

### Survey and data collection

A common European online questionnaire was developed by consensus, and translated into national languages [16]. The questionnaire consists of 53 items divided into six topics: (a) infection prevention; (b) patient flow for COVID-19 and non-COVID-19 care; (c) dealing with new knowledge and protocols; (d) communication with patients; (e) collaboration; (f) wellbeing of the respondent; (g) and characteristics of the respondent and the practice. The Research Electronic Data Capture (REDCap) platform was used to host the questionnaire in all languages, to send out invitations to the national samples of PCPs, and to securely store the answers from the participants at Ghent University. Data were collected by means of an online self-reported questionnaire among PCPs. Sections a, b, c, d and e reflected the whole practice perspective whereas section (f) referred as to personal experience of the respondent, and we have not considered this last part in this study.

### Sampling and recruitment

Sampling was made in each region counting out at least ten practices per region (Supplement 1) for at least, 200 practices nationwide, as recommended by the consortium. Supplement 1 showed the distribution of practices per regions with the average number of healthcare professionals per practice. Researchers’ core team sent five reminder messages to participating PCPs in each region to increase the response rate and make it representative per region as well.

### Analysis

We calculated the prevalence of COVID-19 cases from the beginning of the pandemic until the end of May 2021 (Supplement 1) when the Spanish survey closed (Fig. 1). We liked to assess if the COVID-19 burden of work might influence quality and safety in PCPs. Therefore, we set a prevalence of 5% as cut off point, based on Spanish average burden of COVID-19 estimated in the national SARS-CoV-2 serological survey [15] of summer 2020, which detected a seroprevalence, by the point of care test of 5.0% (95% CI 4.7-5.4). We built two groups of regions. One group with a prevalence less than 5% and the other equal or more than 5%, as there was substantial geographical variability, with higher prevalence around Madrid region (>10%) and lower in coastal areas (<3%).

**Fig. 2:**
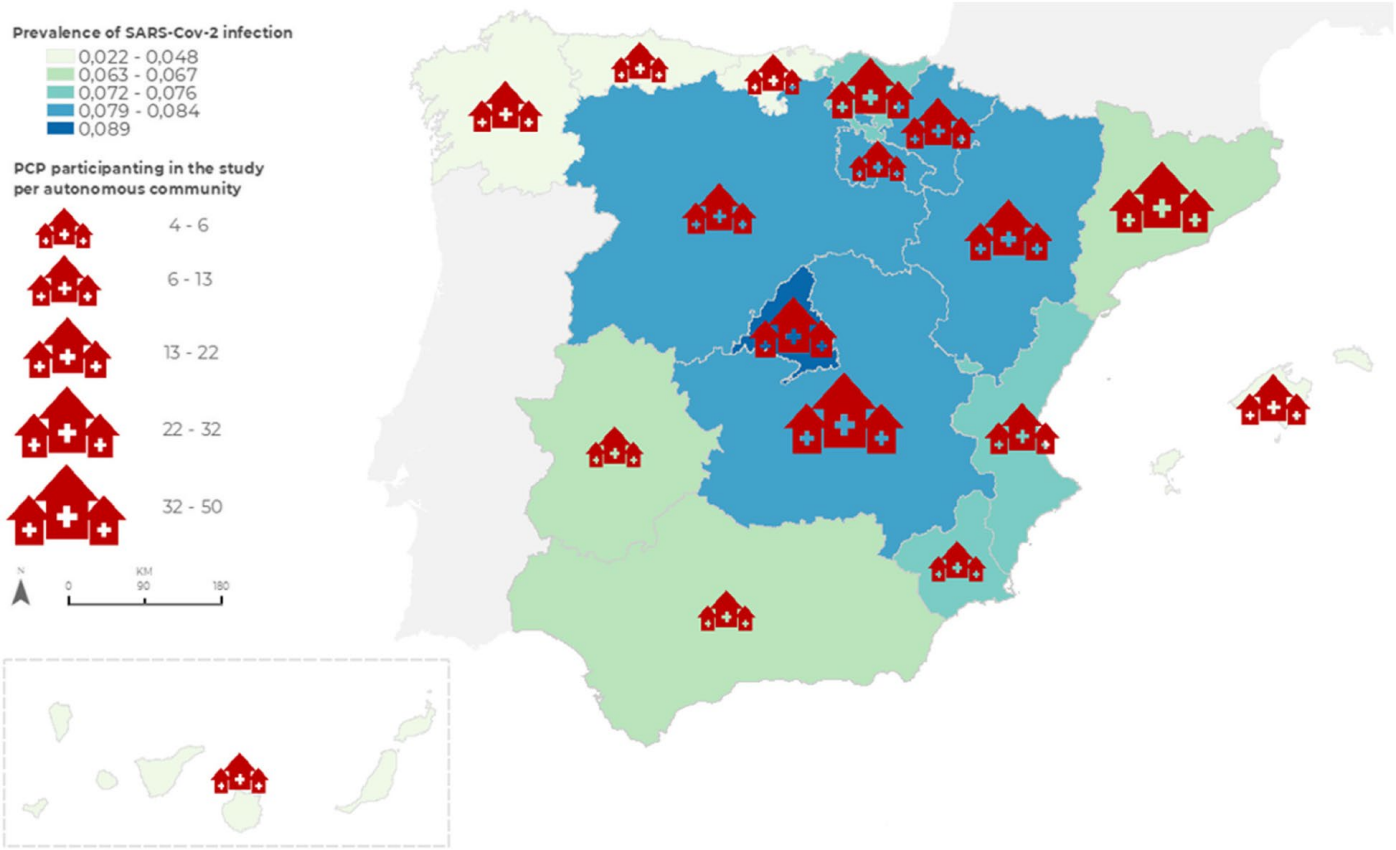
Analysis of the prevalence of SARS-CoV-2 infection based on Autonomous Communities and number of PCPs responding to the questionnaire

We did a descriptive analysis of the items of the questionnaire and made comparisons between the two groups of COVID-19 prevalence (Fig. 2). We performed two logistic regression considering as dependent variable the prevalence of COVID-19. The first regression included structural and administrative pathway variables and the other regarding the COVID-19 clinical pathway variables.

The data analysis was performed with RStudio® 2021.09.0, Stata 16 and the graphics with ArcGIS® Desktop 10.7.1.

## Results

### Descriptive PCPs by COVID-19 prevalence: structural analysis

Two hundred sixty-six PCPs from all the 17 regions of Spain answered the questionnaire completely or nearly completely. Forty-three questionnaires were nearly empty and they were not included in the analysis. Most of PCPs were located in areas with high COVID-19 prevalence (83.8%) compared with those with lower prevalence (16.1%) (Fig. 2). Castilla-León was the region with more answers (n: 55), followed by Cataluña (n: 41) and Madrid region (n: 28). 66.5% of PCPs were in urban areas. Most of the participating PCPs had multi professional teams formed by family doctors (99.6%), nurses (97.4%), administrative assistants (91.7%), social workers (77.1%) and cleaning employee (91.7%). PCPs declared to attend elderly patients (60.9%) and chronic patients (53.0%) above the national average of those types of patients in PHC although the migrant population was below the average (50.8%). More than half of PCPs were teaching practices (62.4%). Considering structural measures, all PCPs fulfilled the safety and hygiene requirements, without statistical differences among higher and lower COVID-19 prevalence areas (Table 1).

**Table 1:** Structural and Administrative Actions according to low and high COVID-19 prevalence regions

		All PHC practices	Prevalence <5%	Prevalence ≥ 5-10%	*p*-value
		(*n*, %)	(*n*, %)	(*n*, %)	
Practice building					
Suitable for the pandemic	None/hardly	45 (16.9)	8 (18.6)	37 (16.6)	0.52
	To a limited extend	95 (35.7)	18 (41.9)	77 (34.5)	
	To a large extend	126 (47.4)	17 (39.5)	109 (48.9)	
Expected renovation work	None/hardly	79 (29.7)	14 (32.6)	65 (29.1)	0.76
	To a limited extend	100 (37.6)	17 (39.5)	83 (37.2)	
	To a large extend	87 (32.7)	12 (27.9)	75 (33.6)	
Role of the government in the practices					
Adequate support	Disagree	181 (68.0)	31 (72.1)	150 (67.3)	0.26
	Neutral	53 (19.9)	10 (23.3)	43 (19.3)	
	Agree	32 (12.0)	2 (4.7)	30 (13.5)	
Guidelines represented a threat to the proper organization Disagree		69 (25.9)	7 (16.3)	62 (27.8)	0.036
Neutral		97 (36.5)	23 (53.5)	74 (33.2)	
Agree		100 (37.6)	13 (30.2)	87 (39.0)	
Cleaning protocols					
Before the pandemic	Never	52 (19.5)	14 (32.6)	38 (17.0)	0.004
	Sometimes	109 (41.0)	21 (48.8)	88 (39.5)	
	Always	105 (39.5)	8 (18.6)	97 (43.5)	
Since the pandemic	Never	13 (4.9)	1 (2.3)	12 (5.4)	0.68
	Sometimes	45 (16.9)	8 (18.6)	37 (16.6)	
	Always	208 (78.2)	34 (79.1)	174 (78.0)	
Administrative actions					
Assistance of patients without an appointment	No	155 (58.3)	25 (58.1)	130 (58.3)	0.98
	Yes	111 (41.7)	18 (41.9)	93 (41.7)	
Patients should explain the reason to get an appointment					
By phone	No	88 (33.1)	25 (58.1)	63 (28.3)	< 0.001
	Yes	178 (66.9)	18 (41.9)	160 (71.7)	
Online	No	224 (84.2)	40 (93.0)	184 (82.5)	0.083
	Yes	42 (15.8)	3 (7.0)	39 (17.5)	
Administrative assistances were more involved in giving information to patients contacting the practice by phone during the pandemic					
Strongly disagree- disagree		26 (9.8)	4 (9.3)	22 (9.9)	0.70
Neutral		35 (13.2)	4 (9.3)	31 (13.9)	
Agree-strongly agree		205 (77.1)	35 (81.4)	170 (76.2)	
Phone protocol for the administrative assistances					
No		125 (47.0)	23 (53.5)	102 (45.7)	0.49
Yes, this protocol is based on a governmental guideline		68 (25.6)	8 (18.6)	60 (26.9)	
Yes, this protocol is not based on a governmental guideline		73 (27.4)	12 (27.9)	61 (27.4)	
Participation of administrative assistances in the triage (phone or in person)					
Disagree		34 (12.8)	7 (16.3)	27 (12.1)	0.72
Neutral		36 (13.5)	5 (11.6)	31 (13.9)	
Agree		196 (73.7)	31 (72.1)	165 (74.0)	
Phone call to verify risk of COVID-19 symptoms before the appointment					
Never/Rarely		101 (38.0)	21 (48.8)	80 (35.9)	0.27
Sometimes		17 (6.4)	2 (4.7)	15 (6.7)	
Usually/Always		148 (55.6)	20 (46.5)	128 (57.4)	
Remote assessment					
Before the pandemic	No	262 (98.5)	43 (100.0)	219 (98.2)	0.38
	Yes	4 (1.5)	0 (0.0)	4 (1.8)	
Since the pandemic	No	218 (82.6)	42 (97.7)	176 (79.6)	0.004
	Yes	46 (17.4)	1 (2.3)	45 (20.4)	
Sending administrative documents to suspicious/confirmed COVID-19					
By e-mail	Never/rarely	63 (23.7)	7 (16.3)	56 (25.1)	0.029
	Sometimes	91 (34.2)	10 (23.3)	81 (36.3)	
	Regularly/always	112 (42.1)	26 (60.5)	86 (38.6)	
By online platform	Never/rarely	157 (59.0)	36 (83.7)	121 (54.3)	< 0.001
	Sometimes	40 (15.0)	5 (11.6)	35 (15.7)	
	Regularly/always	69 (25.9)	2 (4.7)	67 (30.0)	

### Administrative pathways

Administrative assistances were more involved in giving information and recommendations to patients contacting the practice by phone during the pandemic in 77.1% of the practices. Although, 47.0% of the practices didn’t have a phone protocol for the administrative assistances (Table 1). However, administrative assistances asked more frequently to patients what was the main reason to get an appointment in high COVID-19 prevalence areas compared to those with lower prevalence (71.7% vs 41.9%, *p* < 0.001). In suspicious COVID-19 cases, the staff participated in the triage (by phone or face to face) in over 73% of the practices. Patients without an appointment were visited in 41.7% of the practices without differences between high and low prevalence.

Remote assessment was anecdotally before the pandemic with only 4 PCPs (1.5%) offering the service. During the pandemic the service grew till 46 PCPs (17.4%). Practices located in areas with higher prevalence offered more remote assessment than those in lower prevalence areas (20.4% vs 2.3%, p:0.004). However, PCPs in lower prevalence regions were more prone to offer administrative documents by e-mail to suspicious/confirmed COVID-19 cases than those in higher regions (60.5% vs 38.6%, p:0.029). Higher prevalence regions provided online platforms to download administrative documents more frequently than lower ones (30% vs 4.7%, p < 0.001).

### Management of medical care to patients in PCP during COVID-19 pandemic

Accessibility for patients with serious conditions was compromised in 41.0% of the PCPs as patients did not know how to get in touch with a GP (Table 2). Patients with urgent conditions were seen late because they did not consult sooner in 79.1% of the practices in low prevalence area compared to 65.9% of the practices in high prevalence area but not significance was found (p:0.24). Proactive care for chronic patients was provided in 63.5% of the practices. Organizational proactive care through extracting lists from the electronic medical record was run by 35.7% of the practices. People with background of intimate partner violence or mental health issues were scarcely contacted in most of the practices throughout the pandemic. Staff members were more involved in giving information to vulnerable patients (migrants, low health literacy patients or caregivers).

**Table 2:** Characteristics of medical care in PHC practices according to the low and high COVID-19 prevalence regions

		All PHC practices	Prevalence < 5%	Prevalence ≥ 5-10%	*p*-value
		(*n*, %)	(*n*, %)	(*n*, %)	
COVID-19 patients					
Feasibility to isolate	Never/rarely	20 (7.5)	6 (14.0)	14 (6.3)	0.21
	Sometimes	24 (9.0)	4 (9.3)	20 (9.0)	
	Mostly/Always	222 (83.5)	33 (76.7)	189 (84.8)	
Feasibility to attend to the practice/hospital	Never/rarely	12 (4.5)	2 (4.7)	10 (4.5)	0.59
	Sometimes	15 (5.6)	1 (2.3)	14 (6.3)	
	Mostly/Always	239 (89.8)	40 (93.0)	199 (89.2)	
Delay in accessibility					
in patients with a serious condition	No	93 (35.0)	14 (32.6)	79 (35.4)	0.93
	Yes	109 (41.0)	18 (41.9)	91 (40.8)	
	I do not know	64 (24.1)	11 (25.6)	53 (23.8)	
in patients with an urgent condition after phone triage	No	58 (21.8)	6 (14.0)	52 (23.3)	0.24
	Yes	181 (68.0)	34 (79.1)	147 (65.9)	
	I do not know	27 (10.2)	3 (7.0)	24 (10.8)	
Staff members gave information to vulnerable patients^a^	Agree	158 (59.4)	22 (51.2)	136 (61.0)	0.48
	Neutral	68 (25.6)	13 (30.2)	55 (24.7)	
	Disagree	40 (15.0)	8 (18.6)	32 (14.3)	
Proactive care					
Patients that might postpone healthcare	Agree	37 (13.9)	5 (11.6)	32 (14.3)	0.59
	Neutral	59 (22.2)	12 (27.9)	47 (21.1)	
	Disagree	170 (63.9)	26 (60.5)	144 (64.6)	
Patients with chronic conditions	No	97 (36.5)	19 (44.2)	78 (35.0)	0.25
	Yes	169 (63.5)	24 (55.8)	145 (65.0)	
Patients with chronic conditions through lists from the EMR	No	171 (64.3)	28 (65.1)	143 (64.1)	0.90
	Yes	95 (35.7)	15 (34.9)	80 (35.9)	
Patients with background of intimate partner violence	No	180 (67.7)	34 (79.1)	146 (65.5)	0.085
	Yes	31 (11.7)	1 (2.3)	30 (13.5)	
	I do not know	55 (20.7)	8 (18.6)	47 (21.1)	
Patients with background of mental health issues	No	152 (57.1)	31 (72.1)	121 (54.3)	0.071
	Yes	82 (30.8)	10 (23.3)	72 (32.3)	
	I do not know	32 (12.0)	2 (4.7)	30 (13.5)	
Financial problems since the pandemic					
Patients shared	Not at all/less than before	25 (9.4)	2 (4.7)	23 (10.3)	0.11
	As much as before	47 (17.7)	4 (9.3)	43 (19.3)	
	More/much more than before	194 (72.9)	37 (86.0)	157 (70.4)	
GP asked	Not at all/less than before	36 (14.0)	6 (14.3)	30 (13.9)	0.99
	As much as before	131 (50.8)	21 (50.0)	110 (50.9)	
	More/much more than before	91 (35.3)	15 (35.7)	76 (35.2)	

^a^: Vulnerable patients: migrants, low health literacy patients or caregivers

*EMR* Electronic medical record

Feasibility to COVID-19 patients to attend the practice or to isolate themselves was questioned over 89.0% of all the practices. Patients in lower prevalence area talked more about financial problems since the COVID-19 pandemic began than those in high prevalence area (86.0% vs 70-4%, p.0.11). Nevertheless, GPs did not check the financial status more frequently than before the pandemic although GP recognized their role changed in 54.1% of the practices.

### Human resources organization in PCPs during COVID-19 pandemic

COVID-19 sick leaves among healthcare professionals were present in all practices (Table 3). High prevalence regions counted with more backup staff members hired by the health authority to collaborate with the practice activity than in the low prevalence regions. Nurses were the most frequently hired professionals especially in regions with more cases compared with regions with lower cases (63.9% vs 37.8%, *p* < 0.001). Weekly team meetings were reduced since the pandemic started compared with before (55.3% vs 43.2%). In the pandemic, weekly meetings were more frequent in low prevalence regions than in regions of high prevalence.

**Table 3:** Human resources organization in PCPs according to the low and high COVID-19 prevalence regions

		All PHC practices	Prevalence <5%	Prevalence ≥ 5-10%	*p*-value
		(*n*, %)	(*n*, %)	(*n*, %)	
Staff availability					
Staff COVID-19 sick leaves	<=15 persons with sick leaves	221 (83.1)	40 (93.0)	181 (81.2)	0.058
	>15 persons with sick leaves	45 (16.9)	3 (7.0)	42 (18.8)	
Backup staff members					
Nurses	None	70 (27.7)	23 (62.2)	47 (21.8)	< 0.001
	1-3 Nurses	152 (60.1)	14 (37.8)	138 (63.9)	
	≥ 4 nurses	31 (12.3)	0 (0.0)	31 (14.4)	
Medical or Nursing students	None	221 (83.1)	42 (97.7)	179 (80.3)	0.005
	1-4 Students	45 (16.9)	1 (2.3)	44 (19.7)	
Social workers	None	211 (85.4)	37 (100.0)	174 (82.9)	0.006
	1-3 persons	36 (14.6)	0 (0.0)	36 (17.1)	
Administrative assistances	None	107 (42.5)	25 (67.6)	82 (38.1)	0.002
	1-3 persons	128 (50.8)	12 (32.4)	116 (54.0)	
	≥4 persons	17 (6.7)	0 (0.0)	17 (7.9)	
Team meetings					
Before the pandemic	Less than once a week	119 (44.7)	19 (44.2)	100 (44.8)	0.94
	Weekly	147 (55.3)	24 (55.8)	123 (55.2)	
Since the pandemic	Less than once a week	151 (56.8)	18 (41.9)	133 (59.6)	0.031
	Weekly	115 (43.2)	25 (58.1)	90 (40.4)	
Better coordination among PHC practices since the pandemic					
Disagree		180 (67.7)	33 (76.7)	147 (65.9)	0.088
Neutral		53 (19.9)	9 (20.9)	44 (19.7)	
Agree		33 (12.4)	1 (2.3)	32 (14.3)	

The coordination among PCP in the same area did not improve through the pandemic. Buildings were not prepared for the pandemic in many of the practices and two thirds of practices plan to do renovation work. 68% of practices agreed on an inadequate support for the proper functioning of the practice from the government. The role of governmental guidelines on practices because of COVID-19 was negative for 39% of the practices in high prevalence compared to 30.2% of low prevalence (p:0.036).

We performed three regression models regarding structure and COVID-19 clinical management (Table 4). There were no crucial differences among low and high COVID-19 prevalence regions. Only the increasing nursing support was a clear difference among the two compared groups, OR: 4.20 (2.01-8.71).

**Table 4:** Backup staff members and proactive care factors associated with high COD-19 prevalence regions. Characteristics of the PCPs building. (OR and 95% confidence interval for covariates)

	High COVID-19 prevalence regions OR (95% CI)
**Model 1: Backup staff members (reference:low COVID-19 prevalence)**	
Nurses	4.20 (2.01-8.71)
Medical or Nursing students	6.65 (0.84-52.5)
Social workers	0.47 (0.17-1.29)
Administrative assistances	1.83 (0.85-3.94)
**Model 2: Proactive care (reference: low COVID-19 prevalence)**	
Patients that might postpone healthcare	0.93 (0.58-1.48)
Patients with chronic conditions	0.83 (0.44-1.57)
Patients with chronic conditions through lists from the EMR	1.13 (0.55-2.31)
Patients with background of intimate partner violence	0.92 (0.54-1.57)
Patients with background of mental health issues	1.96 (0.99-3.90)
**Model 3: Practice building**	
**Suitable for the pandemic, reference: None/hardly**	
To a limited extend	0.89 (0.34-2.33)
To a large extend	1.29 (0.49-3.39)
**Expected renovation work, reference: None/hardly**	
To a limited extend	1.07 (0.47-2.41)
To a large extend	1.24 (0.51-3.00)

*OR* (95% CI) Odds ratio (95% confidence interval)

## Discussion

There were no big differences in the implementation of initiatives to guarantee the safety and healthcare quality between regions with high and low prevalence of COVID-19 in Spain. Delay in accessibility was found for serious and urgent conditions. Remote assessment was implemented during the pandemic especially in regions with high COVID-19 prevalence. Back-up nurses were hired more frequently in regions with high COVID-19 prevalence.

The lack of differences in most of the variables may be explained because the Spanish Ministry of Health assumed sole command of the pandemic from the declaration of the state of alarm on March 15 until June 21, 2020 [11]. During that period, all regions shared the same protocols to guarantee adequate care for COVID-19 patients based on evidence, the triage procedures, and the use of COVID-19 detection tests in primary care. Subsequently, the ICNHS continued to meet weekly once the autonomy in the management of health services was returned to the regions. Therefore, they continued to make agreements on the care protocols to be applied and the epidemiological information system among all the regions. These meetings facilitated contact between the different regional health services, reducing the variability of COVID-19 care in PCPs. There were differences on the way local health authorities for PHC provided support to PCPs, particularly, to ease connection among health information systems, in the different levels of care to enhance communications technologies and building structures which did not depend upon PCPs but on the PHC regional directorates. As in many countries, virtual care use in primary care saw a transformative change during the pandemic. However, despite the advances in the various governmental guidance offered, much work remains in addressing the shortcomings exposed during COVID-19 and strengthening viable policies to better incorporate novel technologies into the modern primary care clinical environment [17].

Regarding new roles for PHC professionals, administrative assistants had an important role in providing access to medical care especially in high COVID-19 prevalence regions. Nevertheless, in many regions, the local health authority did not provide a phone triage protocol for them. Online appointments were not available in most of the practices whereas administrative documents could be sent by email frequently. Remote assessment grew in regions with higher COVID-19 prevalence, as it happened in other countries as in Israeli. PCPs reported an increase in use of telemedicine from 11 to 49% during the COVID-19 pandemic [18].

The tasks changed in the team assuming different roles to face adequately the pandemic. More back up nurses were hired in high prevalence regions as it happened in other European countries from PRICOV-19 study [19].

While many practices assessed COVID-19 patients to guarantee isolation and practice attendance in case of need, delay in healthcare happened in patients with serious conditions during pandemic and therefore a second pandemic of delayed diagnosis may happen [20]. Among delayed activities were people with background of intimate partner violence or mental health issues that were scarcely contacted in most of the practices throughout the pandemic. In this topic, New Zealand experience [21] indicated that reducing barriers to patients seeking care and improving integration and relationships across the health system would minimise future pandemic disruption and delayed patient healthcare. Nevertheless, proactive care for chronic patients was offered in two thirds of PCPs [22].

Regarding increasing recruitment to support PCPs overload with work, human resources have been paid with the European COVID-19 Fund [23] for Spain, which was equitably distributed according to the population of each region. All regions received a lot of continued reinforcement from the Health Ministry. Spain’s healthcare spending should be growing by over 10.83 billion euros between 2018 and 2030 [24]. One source of increased spending is being the investment in healthcare technology. It will translate into constant average annual spending growth of 2.2%. Spain is expected to allocate additional spending to enhance system interconnectivity, improve patient empowerment, and prevent and monitor chronic conditions. Such e-Health initiatives imply a 1.5% increase in estimated health expenditure. Other regions requiring additional spending include recruiting and retaining healthcare workers as well as the expansion and upgrading of healthcare technology. The additional funds injected help to build a more favourable position for responding to potential future health emergencies including the need to redesign PCPs to adapt them to the pandemic as detected in our study.

### Strengths and limitations

One of the strengths of this study is that the sample size was calculated in advance adjusting for the number of practices in each region of Spain. Although the sampling method was a convenient one, every liaison doctor selected double the size of the sample to increase participation. This survey was collected between the third COVID-19 wave (January 2021) and the fourth wave (March-May 2021) in Spain (Fig. 1). Collecting data in that period allows us to know which changes in the clinical practice were already implemented in addition to describing the difficulties that primary care practices faced. The study included practices in all Spanish regional healthcare systems.

Most of the PCPs were training practices and belonged to economic high-level regions. It might be due that to have training students is a positive stimulus for a better performance as it is stated in PRICOV-19 analysis on the impact of being training practices [25]. Training practices were found to have a stronger safety culture than non-training practices. These results have important policy implications, since involving more PCPs in education may be an effective way to improve quality and safety in general practice.

Several limitations should be taken in account. Firstly, the questionnaires were self-reported with information about the whole practice. This information could be influenced by the personal point of view of the respondent, and it could not be representative of all the PCP Team. Secondly, the participation was voluntary. Although the researchers sent 5 reminder messages, the circumstance of overwork in PCP due to new waves of pandemic during the study period (from January to May 2021) we failed to increase the response rate to make it representative per region.

An overrepresentation of practices who were more engaged with the patients could be found, as an example, a total of 62% of PCP sampling were teaching practices. Thirdly, the survey was fully completed in 244 practices out of 266 and partially in 22 practices. Partial answers included at least half of the survey completed.

### Implications for practice

A national health system with a single command from the Spanish Health Ministry allowed to provide standardized healthcare for COVID-19 patients in the whole country during the first wave of the pandemic. Although the healthcare was decentralized afterwards few changes were found among the regions. It could be important to keep this centralized organization to avoid healthcare differences to COVID-19 patients among the regions but this organization did not provide resources to improve care to non-COVID-19 patients or vulnerable patients.

In Spain and in the EU, there is a lack of a national primary care contingency plan to standardize a minimum set of actions to tackle a pandemic. A contingency plan should be supported with a budget to run primary care with enough resources to adapt to new situations. It is essential to design a more structured pathway to treat non-COVID-19 patients during the pandemic to avoid delays in serious diseases. Although, remote assessment improved during the pandemic, more efforts are needed to generalise the use of these tools (online appointments, online interviews, and online platforms to download data from the electronic health record). More staff was hired but it was not enough to avoid the delay in medical care. This must be understood under the under-resourced personnel in primary care in Spain. There is an urgent need to expand the staff in the practices, especially the administrative assistances and nurses. A public health officer in each practice to surveillance COVID-19 could be a useful role to incorporate in primary teams.

## Conclusions

Spanish PCPs shared a basic standardized PCPs’ structure and common main clinical procedures due to the centralization of Public Health Authority in the pandemic. Therefore, no relevant differences in safety and quality of care between regions with high and low prevalence was detected. Nurses and administrative staff were hired efficiently in response to the pandemic. Delay in care happened in patients with serious conditions and scarce follow-up for mental health and intimate partner violence affected patients was identified. Nevertheless, proactive care was offered for chronic patients in most of the PCPs.

### Supplementary Information

Supplementary Material 1: Primary Healthcare Teams in Spanish National Health Services, December 2020.

## Data Availability

All data are centrally stored on the server of Ghent University (Belgium). All data was anonymized at Ghent University, and all raw data that could lead to the identification of the respondents was permanently removed. Reasonable request is required to access non-identifiable data by users who are external to the PRICOV-19 consortium. Access will be subject to a data transfer agreement and following approval from the principal investigator of the PRICOV-19 study.

## Abbreviations

PHC: Primary Health Care
PCT: Primary Health Care Teams
PCP: Primary Health Care Practices or Health Centers
FD: Family Doctor
SNHS: Spanish National Healthcare System
ICSNHS: Inter-territorial Committee for the National Healthcare System
REDCap: Research Electronic Data Capture
