# Supplementary material for: Quality and safety actions in primary care practices in COVID-19 pandemic: the PRICOV-19 study in Spain
Source: BMC Prim Care. 2024 May 13;24(Suppl 1):286. doi: 10.1186/s12875-024-02391-8 (PMC11089663; doi:10.1186/s12875-024-02391-8)
Supplement: Supplementary file 1 — Supplementary Material 1: Primary Healthcare Teams in Spanish National Health Services, December 2020. [file 12875_2024_2391_MOESM1_ESM.docx]

Supplement 1: Primary Healthcare Teams in Spanish National Health Services, December 2020

| **Autonomous Community** | **N of Health Centers** | **Professional Category** | **N of Professionals** | **Professional average per center** | **Professional Sampling** | **Health Center Sample** |
| --- | --- | --- | --- | --- | --- | --- |
| **ANDALUCÍA** | **407** | **Family Medicine** | 4.992 | 36,23 |  |  |
|  |  | **Paediatrics** | 1.152 |  |  |  |
|  |  | **Nursing** | 4.726 |  |  |  |
|  |  | **Nursing Assistant** | 1.231 |  |  |  |
|  |  | **Administrative Assistant** | 2.645 |  |  |  |
|  |  | **Total** | 14.746 |  | 200 | 10 |
| **ARAGÓN** | **118** | **Family Medicine** | 967 | 21,56 |  |  |
|  |  | **Paediatrics** | 182 |  |  |  |
|  |  | **Nursing** | 961 |  |  |  |
|  |  | **Nursing Assistant** | 79 |  |  |  |
|  |  | **Administrative Assistant** | 355 |  |  |  |
|  |  | **Total** | 2.544 |  | 188 | 10 |
| **ASTURIAS (PRINCIPADO DE)** | **69** | **Family Medicine** | 670 | 28,97 |  |  |
|  |  | **Paediatrics** | 126 |  |  |  |
|  |  | **Nursing** | 761 |  |  |  |
|  |  | **Nursing Assistant** | 80 |  |  |  |
|  |  | **Administrative Assistant** | 362 |  |  |  |
|  |  | **Total** | 1.999 |  | 184 | 7 |
| **BALEARES (ISLAS)** | **58** | **Family Medicine** | 551 | 28,55 |  |  |
|  |  | **Paediatrics** | 142 |  |  |  |
|  |  | **Nursing** | 568 |  |  |  |
|  |  | **Nursing Assistant** | 101 |  |  |  |
|  |  | **Administrative Assistant** | 294 |  |  |  |
|  |  | **Total** | 1.656 |  | 181 | 8 |
| **CANARIAS** | **106** | **Family Medicine** | 1.194 | 45,73 |  |  |
|  |  | **Paediatrics** | 320 |  |  |  |
|  |  | **Nursing** | 1.836 |  |  |  |
|  |  | **Nursing Assistant** | 309 |  |  |  |
|  |  | **Administrative Assistant** | 1.188 |  |  |  |
|  |  | **Total** | 4.847 |  | 195 | 8 |
| **CANTABRIA** | **42** | **Family Medicine** | 368 | 26,95 |  |  |
|  |  | **Paediatrics** | 84 |  |  |  |
|  |  | **Nursing** | 432 |  |  |  |
|  |  | **Nursing Assistant** | 56 |  |  |  |
|  |  | **Administrative Assistant** | 192 |  |  |  |
|  |  | **Total** | 1.132 |  | 172 | 8 |
| **CASTILLA Y LEÓN** | **247** | **Family Medicine** | 2.312 | 22,03 |  |  |
|  |  | **Paediatrics** | 252 |  |  |  |
|  |  | **Nursing** | 2.106 |  |  |  |
|  |  | **Nursing Assistant** | 156 |  |  |  |
|  |  | **Administrative Assistant** | 616 |  |  |  |
|  |  | **Total** | 5.442 |  | 196 | 8 |
| **CASTILLA-LA MANCHA** | **204** | **Family Medicine** | 1.350 | 18,15 |  |  |
|  |  | **Paediatrics** | 237 |  |  |  |
|  |  | **Nursing** | 1.456 |  |  |  |
|  |  | **Nursing Assistant** | 63 |  |  |  |
|  |  | **Administrative Assistant** | 596 |  |  |  |
|  |  | **Total** | 3.702 |  | 192 | 10 |
| **CATALUÑA** | **424** | **Family Medicine** | 4.594 | 35,68 |  |  |
|  |  | **Paediatrics** | 1.030 |  |  |  |
|  |  | **Nursing** | 5.398 |  |  |  |
|  |  | **Nursing Assistant** | 638 |  |  |  |
|  |  | **Administrative Assistant** | 3.469 |  |  |  |
|  |  | **Total** | 15.129 |  | 200 | 10 |
| **COMUNIDAD VALENCIANA** | **285** | **Family Medicine** | 2.797 | 31,77 |  |  |
|  |  | **Paediatrics** | 809 |  |  |  |
|  |  | **Nursing** | 2.969 |  |  |  |
|  |  | **Nursing Assistant** | 759 |  |  |  |
|  |  | **Administrative Assistant** | 1.721 |  |  |  |
|  |  | **Total** | 9.055 |  | 200 | 10 |
| **EXTREMADURA** | **111** | **Family Medicine** | 817 | 20,04 |  |  |
|  |  | **Paediatrics** | 127 |  |  |  |
|  |  | **Nursing** | 904 |  |  |  |
|  |  | **Nursing Assistant** | 78 |  |  |  |
|  |  | **Administrative Assistant** | 298 |  |  |  |
|  |  | **Total** | 2.224 |  | 190 | 10 |
| **GALICIA** | **398** | **Family Medicine** | 1.854 | 12,91 |  |  |
|  |  | **Paediatrics** | 307 |  |  |  |
|  |  | **Nursing** | 1.809 |  |  |  |
|  |  | **Nursing Assistant** | 218 |  |  |  |
|  |  | **Administrative Assistant** | 949 |  |  |  |
|  |  | **Total** | 5.137 |  | 195 | 10 |
| **MADRID (COMUNIDAD DE)** | **262** | **Family Medicine** | 3.627 | 38,97 |  |  |
|  |  | **Paediatrics** | 923 |  |  |  |
|  |  | **Nursing** | 3.319 |  |  |  |
|  |  | **Nursing Assistant** | 393 |  |  |  |
|  |  | **Administrative Assistant** | 1.948 |  |  |  |
|  |  | **Total** | 10.210 |  | 200 | 10 |
| **MURCIA (REGIÓN DE)** | **85** | **Family Medicine** | 836 | 28,69 |  |  |
|  |  | **Paediatrics** | 243 |  |  |  |
|  |  | **Nursing** | 817 |  |  |  |
|  |  | **Nursing Assistant** | 115 |  |  |  |
|  |  | **Administrative Assistant** | 428 |  |  |  |
|  |  | **Total** | 2.439 |  | 187 | 10 |
| **NAVARRA (COMUNIDAD FORAL DE)** | **58** | **Family Medicine** | 402 | 22,34 |  |  |
|  |  | **Paediatrics** | 106 |  |  |  |
|  |  | **Nursing** | 507 |  |  |  |
|  |  | **Nursing Assistant** | 1 |  |  |  |
|  |  | **Administrative Assistant** | 280 |  |  |  |
|  |  | **Total** | 1.296 |  | 175 | 7 |
| **PAÍS VASCO** | **154** | **Family Medicine** | 1.469 | 27,62 |  |  |
|  |  | **Paediatrics** | 340 |  |  |  |
|  |  | **Nursing** | 1.558 |  |  |  |
|  |  | **Nursing Assistant** | 128 |  |  |  |
|  |  | **Administrative Assistant** | 759 |  |  |  |
|  |  | **Total** | 4.254 |  | 194 | 10 |
| **LA RIOJA** | **20** | **Family Medicine** | 216 | 32,3 |  |  |
|  |  | **Paediatrics** | 44 |  |  |  |
|  |  | **Nursing** | 290 |  |  |  |
|  |  | **Nursing Assistant** | 35 |  |  |  |
|  |  | **Administrative Assistant** | 61 |  |  |  |
|  |  | **Total** | 646 |  | 154 | 5 |
| **CEUTA y MELILLA (INGESA)** | **7** | **Family Medicine** | 70 | 30,86 |  |  |
|  |  | **Paediatrics** | 24 |  |  |  |
|  |  | **Nursing** | 82 |  |  |  |
|  |  | **Nursing Assistant** | 7 |  |  |  |
|  |  | **Administrative Assistant** | 33 |  |  |  |
|  |  | **Total** | 216 |  | 123 | 4 |
